# Strategies utilized by plants to defend against *Ralstonia solanacearum*

**DOI:** 10.3389/fpls.2025.1510177

**Published:** 2025-05-26

**Authors:** Dexing Xue, Weifeng Wu, Danyu Kong

**Affiliations:** Jiangxi Provincial Key Laboratory of Plant Germplasm Resources Innovation and Genetic Improvement, Lushan Botanical Garden, Chinese Academy of Sciences, Jiujiang, China

**Keywords:** *Ralstonia solanacearum*, inducible defense, cell wall integrity, plant structural barrier, vascular pathogen, bacterial wilt

## Abstract

*Ralstonia solanacearum*, the causal agent of bacterial wilt, is recognized as one of the most destructive vascular pathogens. Plant defense responses are gradually developed through long-term interactions with *R. solanacearum*. The plant cell wall integrity (CWI) system has evolved to initiate defense responses via a diverse array of plasma membrane-resident sensors. These defense responses result primarily from physical and chemical actions that counteract infection with *R. solanacearum*. The plant cell wall serves as a defensive barrier against the pathogen, including cellulose, hemicellulose, pectin, lignin, and suberin. Various modifications to the cell wall and multiple changes in its composition are employed by plants resistant to *R. solanacearum*. Physical confinement vertically or horizontally induced in xylem tissues is the most effective method of defense against *R. solanacearum*. The timely formation of tyloses and gels within the vessel lumen contributes to the suppression of *R. solanacearum*. In addition, the deposition of callose at the infected sites reinforces the cell wall, thereby preventing the further spread of *R. solanacearum*. Morphological modifications, such as the thickening of the pit membranes and the increased number of larger xylem vessels, play crucial roles in conferring resistance to *R. solanacearum*. Secondary metabolites act as phytoalexins used by plants against *R. solanacearum*. In this review, we discuss the strategies deployed by plants resistant to *R. solanacearum*. In particular, we outline the physical and chemical restrictions, as well as the tissue constraints, against the vascular pathogen.

## 1 Introduction

Soil-borne pathogens are a significant cause of crop losses in agricultural species, posing a threat to global agriculture and food security (McCann, 2020). As the causal agent of bacterial wilt, *Ralstonia solanacearum* is in the list of the most scientifically significant plant pathogens (Mansfield et al., 2012). This pathogen has an extremely broad host range and infects more than 250 plant species, including tomato, tobacco, potato, eggplant, and peanut (Genin, 2010; Genin and Denny, 2012; Peeters et al., 2013; Xiao et al., 2023). The *R. solanacearum* species complex (RSSC) comprises three distinct species—*R. solanacearum*, *Ralstonia pseudosolanacearum*, and *Ralstonia syzygii—*all of which share a core genome (Paudel et al., 2020). Among the virulent determinants of *R. solanacearum*, swimming/motility, the cell wall-degrading enzymes (CWDEs), the type III secretion system (T3SS), and exopolysaccharide (EPS) are critical for its pathogenicity (Valls et al., 2006; Milling et al., 2011; Coll and Valls, 2013; Vailleau and Genin, 2023). The bacteria gain entry into the root cortex through the root tips, wounds, or cracks at sites of lateral root emergence and subsequently invade the xylem vessels (Vailleau et al., 2007; Digonnet et al., 2012). *R. solanacearum* in the xylem vessels proliferate up to high cell densities, ultimately disrupting the water conductance and inducing wilting symptoms (Xue et al., 2020). Plants are persistently challenged by *R. solanacearum* during their entire growth period, but develop an innate immune system to counteract the threat (Martin et al., 2003; Jones and Dangl, 2006). The innate immune system of plants consists of two layers (Chisholm et al., 2006; Boller and Felix, 2009). The first layer is known as pathogen-associated molecular pattern (PAMP)-triggered immunity (PTI), which recognize PAMPs by corresponding pattern recognition receptors (PRRs) in the plasma membrane. Recognition by plant PRRs initiates the downstream defense responses such as the production of reactive oxygen species (ROS), cytosolic Ca^2+^ burst, the activation of mitogen-activated protein kinases (MAPKs), and the expression of defense-related genes (Dodds and Rathjen, 2010; Zipfel, 2014). The other layer is effector-triggered immunity (ETI), which is based on the direct or indirect recognition of specific effectors by resistant proteins containing nucleotide-binding leucine-rich repeats (NB-LRRs). ETI produces faster, longer, and stronger responses than PTI, thereby quickly triggering a hypersensitive response (HR), i.e., an induced cell death (Stuart et al., 2013; Huet, 2014). ETI cooperates with PTI to protect plants from pathogenic attacks (Ngou et al., 2021). The two-layer immune system of plants initiates a series of resistant responses at the cellular and tissue levels (Planas-Marquès et al., 2020; Shi et al., 2023). The regulatory responses are involved in tissue constraints, modifications of the cell wall, inducible defenses, and resistant metabolites to counteract the invasion of *R. solanacearum* (Kashyap et al., 2021; Shi et al., 2023). This review focuses on a summary of the strategies deployed by plants resistant to *R. solanacearum*, but also provides an overview of the regulatory mechanisms against bacterial wilt disease.

## 2 Different tissue constraints deployed by plants

**Figure 1:**
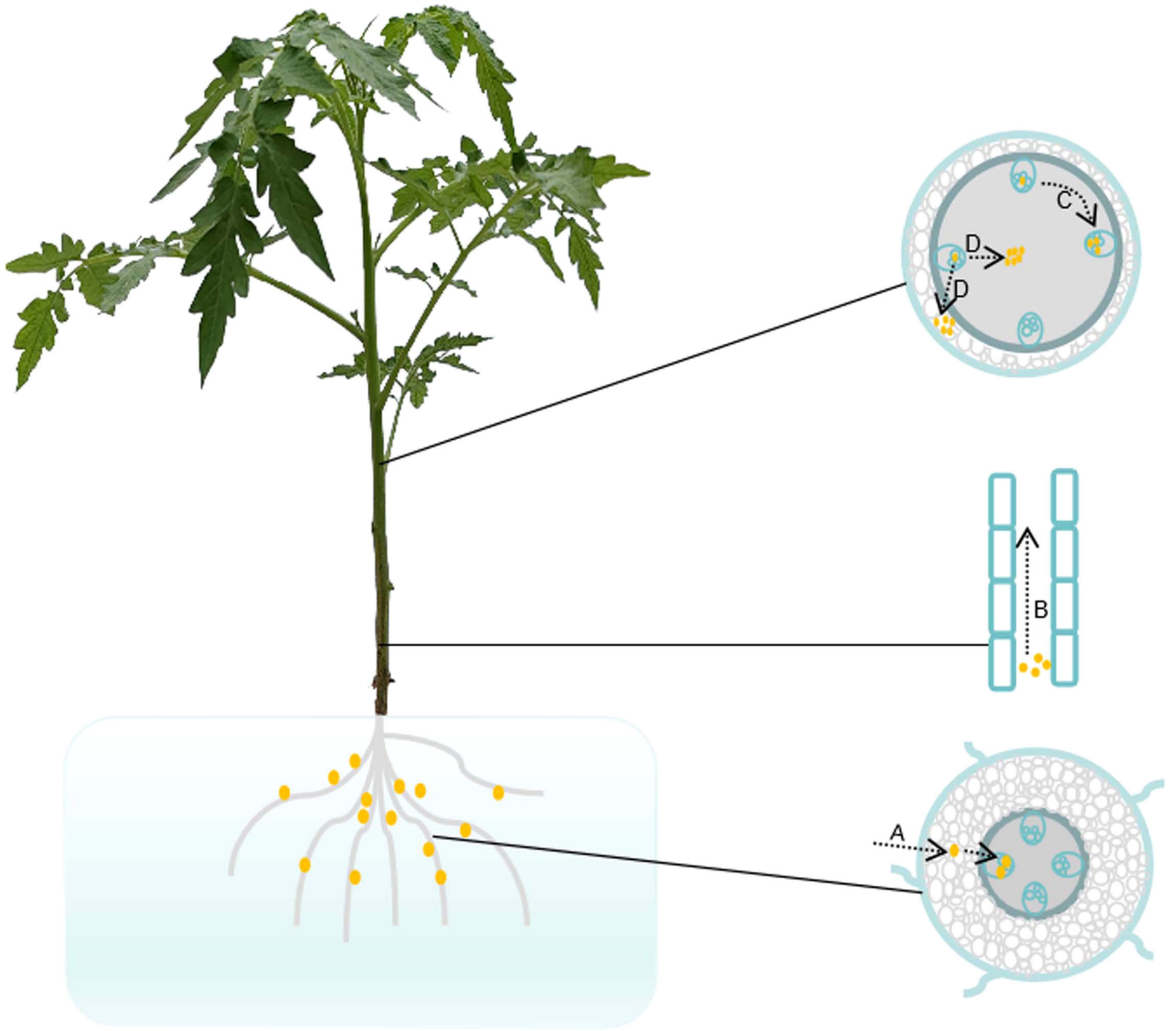
Pathogenic progress of *Ralstonia solanacearum* in tomato plant. **(A)** Tomato roots infected with *R. solanacearum*. *R. solanacearum* enters the root cortex through the tips, wounds, or cracks at infected sites, subsequently invading and proliferating within the root vasculature. **(B)** Vertical movement of *R. solanacearum* upward to the stem. *R. solanacearum* in the roots spreads vertically up to the stem vascular bundles. **(C)** Circular passage of *R. solanacearum* between xylem vessels. *R. solanacearum* in the xylem ducts moves circularly and proliferates among the xylem vessels. **(D)** Radial/apoplastic movements of *R. solanacearum* into the pith/cortex tissue. *R. solanacearum* spreads in the intercellular spaces, multiplies out of the xylem vessels into the pith and cortex, and finally causes the plant to wilt. *Brown dots* represent *R. solanacearum*.

*R. solanacearum* infects the roots through wounds or natural openings and rapidly multiplies in the xylem vessels (Schell, 2000; Mansfield et al., 2012). The bacteria accumulate in xylem ducts, potentially obstructing the water flow and eventually causing the plant to wilt (Hikichi et al., 2017; Mori et al., 2018). Grafting tests have confirmed that Hawaii 7996 rootstock is able to restrict *R. solanacearum* up to the stem in tomato (Nakaho et al., 2004; Truong et al., 2015). In susceptible tomato roots, *R. solanacearum* diffuses more rapidly from the cortex to the vascular system when compared with resistant Hawaii 7996 plants (Caldwell et al., 2017). Upon infection with *R. solanacearum*, petunia forms more lateral root structures. However, these elongated lateral roots do not contribute to resistance against the pathogen (Zolobowska and Van Gijsegem, 2006). Resistance to *R. solanacearum* relies on four different steps (**Figure 1**), which limit the bacterial spread: i) invasion of the plant root; ii) vertical movement upward to the stem; iii) circular passage from vessel to vessel; and iv) radial movement from xylem vessels into the pith/cortex (Planas-Marquès et al., 2020). Morphological changes between susceptible and resistant roots are observed upon infection with *R. solanacearum* (Xue et al., 2020). Primary root growth is significantly inhibited after infection with *R. solanacearum* (Digonnet et al., 2012; Xue et al., 2020). An increased number of larger xylem vessels within resistant roots are observed when compared with susceptible plants via histological staining (Caldwell et al., 2017). It is credible that the larger diameter of xylem vessels impedes bacterial colonization. The ability to limit *R. solanacearum* spreading up to tobacco stem is one of the effective strategies used by plants resistant to the pathogen (Bittner et al., 2016). The resistant rootstock cultivar LS-89, which limits the movement of *R. solanacearum* between xylem vessels, exhibits thickened pit membranes (Nakaho et al., 2000). *R. solanacearum*-resistant rootstocks promote a significant effect on yield in field trials compared with the non-grafted “BHN 602” (McAvoy et al., 2012). Bioluminescence imaging arrays have demonstrated that the colonization and multiplication of *R. solanacearum* are confined within plant roots; however, a limited number of bacteria are detected in stem ducts (Ferreira et al., 2017). After inoculation with *R. solanacearum*, *Solanum dulcamara* exhibits delayed symptomatology; moreover, the bacterial progression is notably restricted within the roots (Sebastià et al., 2021). The distinct morphology of the stem plays vital roles in limiting bacterial colonization and movement (Planas-Marquès et al., 2020). Once *R. solanacearum* penetrates the vascular cylinder of susceptible plants, these bacteria are able to proliferate rapidly (Shi et al., 2023). When the *R. solanacearum* populations reach 5 × 10^8^ CFU/g, approximately half of the xylem vessels from the stem are clogged, thus correlating with the onset of wilt symptoms (Ingel et al., 2022). When compared with the susceptible cultivar Ponderosa, *R. solanacearum* is merely observed in the primary xylem tissues and less in the secondary xylem of resistant LS-89 plants (Nakaho et al., 2000; Ishihara et al., 2012). In addition, thickening of the pit membranes is observed in the LS-89 stems using scanning electron microscopy (Nakaho et al., 2000; Ishihara et al., 2012). These studies demonstrate that morphological constraints contribute to resistance to *R. solanacearum*.

## 3 Structural barriers induced by *R. solanacearum*

**Figure 2:**
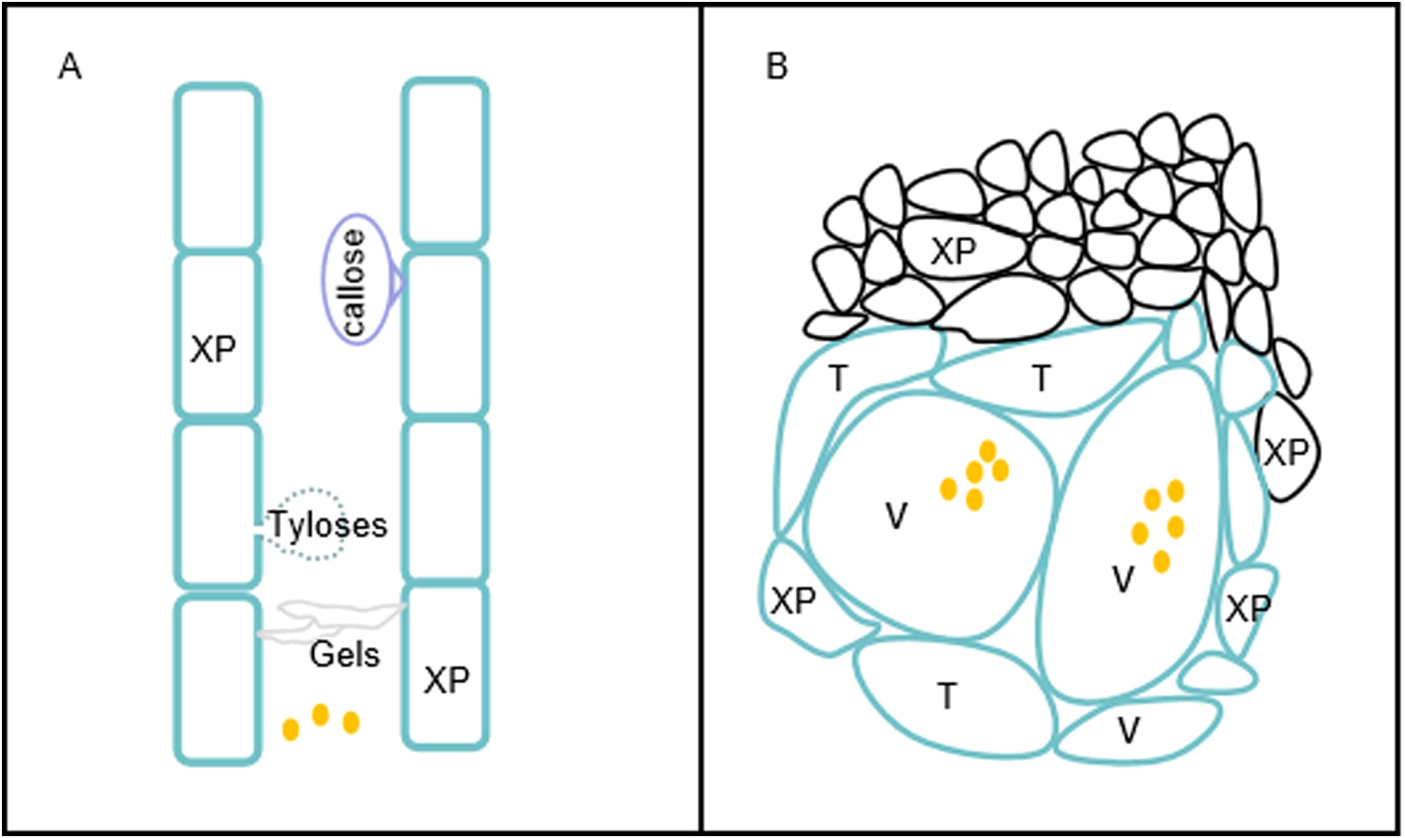
Physical barriers induced by *Ralstonia solanacearum*. **(A)** Vertical restriction of the movement of *R. solanacearum* in the xylem duct. Resistant plants produce tyloses, gels, and callose that restrict the vertical movement of *R. solanacearum*. **(B)** Horizontal restriction of *R. solanacearum* spread in xylem vessels. Vascular coating with lignin and suberin and reinforcement of the xylem vessels (*V*), the surrounding xylem parenchyma (*XP*), and tracheids (*T*) impede the horizontal spread of *R. solanacearum* to the surrounding tissues. *Brown dots* represent *R. solanacearum*.

The number of *R. solanacearum* in the xylem vessels of plants increase up to a high density, subsequently causing plant wilting. The vascular barriers induced by *R. solanacearum* comprise one of the important defenses against bacterial wilt disease. Among the inducible defenses, we focused on the formation of tyloses and the deposition of gels and callose against the invasion of *R. solanacearum* (**Figure 2A**). Parenchyma cells protrude into the xylem vessels through the pit membranes and form tyloses to limit the spread of the pathogen. In contrast, gels and callose are deposited in the xylem vessels in response to pathogenic invasion. The tyloses and gels occlude vascular vessels to restrict the vertical progression of *R. solanacearum*. The timely formation of physical barriers upon pathogenic perception results in the confinement of *R. solanacearum* at the infected vessel and effectively prevents bacterial movement.

### 3.1 Formation of tyloses

Tyloses are balloon-like structures of parenchyma cells that grow out into the lumen of the xylem vessels (Lowe-Power et al., 2018). These outgrowths form a physical barrier and prevent *R. solanacearum* from spreading. In order to prevent the pathogenic spread, the tyloses in tomato varieties are induced at the infected sites (Grimault et al., 1994; Leśniewska et al., 2017). A lower density of tyloses is found in *R. solanacearum*-susceptible tomato plants using light and electron microscopy (Grimault et al., 1994). In addition, the formation of tyloses in susceptible stems is significantly delayed (Grimault et al., 1994). However, extensive formation of tyloses in Pierce’s disease (PD)-susceptible grapevines occludes the xylem vessels and impairs water conductance (Sun et al., 2013). Furthermore, the tyloses in PD-resistant grapevines develop specifically and emerge at the sites of inoculation (Sun et al., 2013). The process of tylosis formation is precisely and tightly regulated by hormones such as auxin, ethylene (ET), and jasmonate (JA) (Kashyap et al., 2021; Shi et al., 2023). Grapevine plants treated with ET exhibit an increased density of tyloses, suggesting that ET is required for tylosis formation (Pérez-Donoso et al., 2007). Similarly, ET inhibitors abolish tylosis formation in grape stems, suggesting that ET is essential for the formation of tyloses (Sun et al., 2008). JA acts synergistically with ET to promote tylosis formation (Leśniewska et al., 2017). These findings suggest that the timely formation of tyloses is essential for resistance against pathogenic attacks. Wide xylem vessels that allow for concentrated tyloses are less efficient in forming compartmentalization, thereby conferring susceptibility of grapevine to the vascular pathogen *Phaeomoniella chlamydospora* when compared with xylem vessels with a narrow diameter (Pouzoulet et al., 2017). In addition to pathogenic invasion, tyloses are formed in response to abiotic stresses, such as freezing and wounding (Kashyap et al., 2021; Shi et al., 2023). Tyloses produce organs of antimicrobial compounds in addition to serving as a structural barrier. Compounds in the tyloses of tomato plants such as elemental S, which is detected using gas chromatography–mass spectroscopy (GC-MS), act as fungicides to inhibit spore germination of the fungal pathogen *Verticillium dahliae* (Williams et al., 2002).

### 3.2 Deposition of gels

The deposition of gels within the lumen of xylem vessels is observed during the invasion of vascular pathogens and functions as an inducible defense (Shi et al., 2023). The secretion of gels in xylem vessels often coincides with the formation of tyloses. Pectin constitutes the primary component of the gels, which contain antimicrobial compounds such as elemental sulfur and phytoalexins (Clérivet et al., 2000; Williams et al., 2002). Gels appear as translucent fibers and contain continuous pectin-rich substances that obstruct the xylem vessels (Sun et al., 2008). Both xylem parenchyma cells and tyloses secrete the gels, which are then transported through pit membranes into vessel elements (Bishop and Cooper, 1984; Sun, 2022). Furthermore, the gels are strengthened by the cross-linking of lignin and phenolic compounds, thereby forming strong physical barriers that impede the movement of *R. solanacearum* (Kashyap et al., 2021). Vascular gels induced by *R. solanacearum* are associated with the defense response (Kashyap et al., 2021). The formation of vascular gels is induced in *R. solanacearum*-resistant tomato cultivars, suggesting a correlation with resistance to *R. solanacearum* (Bishop and Cooper, 1984; Kim et al., 2016).

### 3.3 Deposition of callose

Callose is composed of hundreds of polysaccharides linked by β-1,3 glycosidic bond (Kashyap et al., 2021). The homopolysaccharide that is deposited between the plasma membrane and the cell wall plays a crucial role in biological processes such as plant development and stress response. Callose strengthens the cell wall structure by increasing its rigidity at the infected site and by diminishing the activity of pathogen-secreted CWDEs (Wang et al., 2021). Wang et al. (2021) speculated that the deposition of callose acts as one of the early defense responses of plants against pathogenic invasion (Wang et al., 2021). Numerous studies have shown that callose deposition is correlated with the defense of plants against bacterial, fungal, and viral infection. Upon treatment with bacterial pathogens such as *Xanthomonas campestris* pv. *vesicatoria* and *Pseudomonas syringae* pv. *phaseolicola*, callose is deposited to reinforce the plant cell wall at the infected sites (Bestwick et al., 1995; Brown et al., 1998; Wang et al., 2021). Callose is also induced by PAMPs including flagellin (flg22), elongation factor Tu (EF-Tu; elf18), chitin, and chitosan (Couto and Zipfel, 2016; Zipfel and Oldroyd, 2017). Barley papillae, which contain callose and high concentrations of cellulose, serve as physical barriers preventing the penetration of the fungus *Blumeria graminis* f. sp. *hordei* (Chowdhury et al., 2014). During the incompatible interaction between soybean and soybean mosaic virus (SMV), callose is deposited in the plasmodesmata to restrict viral movement between cells (Li et al., 2012). The exogenous application of salicylic acid (SA) induces callose deposition in the plasmodesmata, indicating that SA plays a crucial role in the deposition of callose (Wang et al., 2013). Potato plants overexpressing NACb4 are capable of inducing callose deposition and enhancing tolerance against *R. solanacearum* (Chang et al., 2020). The endophytic bacterium has the potential to prime callose deposition upon *R. pseudosolanacearum* GMI1000 infection (Rodriguez et al., 2019). It was found that *R. solanacearum-*resistant potatoes display similar callose deposition density to plants not inoculated with the pathogen, suggesting that the callose preexists in resistant plants (Ferreira et al., 2017). Ferreira et al. (2017) proposed that the preexisting callose is deposited into the cell wall of plants resistant to *R. solanacearum*.

## 4 Alterations of plant cell wall resistant to *R. solanacearum*

The plant cell wall serves as a crucial barrier against pathogenic invasion (Malinovsky et al., 2014). Pathogens secrete CWDEs that hydrolyze the linkages between glycan moieties, thereby breaking down the barrier. The development of the plant cell wall is a dynamic process by which the synthesis and modifications are integrated to regulate the resistance to pathogens (Bacete et al., 2018). The composition of the plant cell wall that is integral to the defense against pathogens mainly comprises cellulose, hemicellulose, pectin, lignin, and suberin (Shi et al., 2023). The plant cell wall integrity (CWI) has a significant effect on abiotic and biotic stresses (Miedes et al., 2014; Kesten et al., 2017; Bacete et al., 2018). Modifications to the cell wall have been shown to affect pathogen resistance (Malinovsky et al., 2014). Plants have evolved a specialized mechanism to maintain CWI, thereby providing effective resistance to diseases (Bellincampi et al., 2014; Malinovsky et al., 2014). When the CWI of plants is compromised, the CWI system monitors the state of the cell wall and subsequently activates innate immune responses (Shi et al., 2023). The oligogalacturonides (OGAs) derived from pectic homogalacturonan (HGA) are recognized by wall-associated kinases (WAKs) that sense the integrity of pectin (Ferrari et al., 2013). The xylem vessels of resistant plants exhibit reinforced pit membranes, thereby impeding the movement of pathogens between vessels and the vessel/parenchyma (Choat et al., 2008; Sun et al., 2011; Kashyap et al., 2021). Reinforcement of the cell wall is able to limit the horizontal movement of *R. solanacearum* between xylem vessels (Kashyap et al., 2021). Moreover, the composition and the structure of xylem pit membranes are altered in resistant plants (Kashyap et al., 2021). When compared with PD-susceptible plants, the pit membranes of resistant grapevine are found to lack fucosylated xyloglucans and weakly methyl-esterified homogalacturonans (ME-HGs) and to encompass a small amount of heavily ME-HGs (Sun et al., 2011).

Cellulose, which is synthesized by plasma membrane-localized cellulose synthase complexes (CSCs), plays an important role in the defense against pathogenic attacks (Polko and Kieber, 2019). Each unit of the CSC is composed of at least three different cellulose synthases (CESAs) (Somerville, 2006). CESA1, CESA3, and CESA6 are required in the formation of cellulose in primary walls, while CESA4, CESA7, and CESA8 are responsible for the production of secondary wall cellulose (Hernandez-Blanco et al., 2007; Endler and Persson, 2011). *Arabidopsis* mutants deficient in CESA4/7/8 exhibit increased resistance to *R. solanacearum* (Hernandez-Blanco et al., 2007; Wan et al., 2021). The loss of function of MYB46, which positively regulates the expression of CESA4/7/8, enhances the resistance of *Arabidopsis* plants to *Botrytis cinerea* (Wan et al., 2021). There are a large number of cases showing that the inhibition of cellulose synthesis results in increased susceptibility to plant diseases (Shi et al., 2023). When cellulose synthase-like D2 is silenced, transgenic plants show enhanced susceptibility to powdery mildew (Douchkov et al., 2016). The transcription factor WRKY53 promotes the expression of three secondary cell wall-related cellulose synthase genes, thereby conferring rice resistance to *Xanthomonas oryzae* pv. *oryzae* (*Xoo*) by strengthening the sclerenchyma cell walls surrounding the xylem vessel (Xie et al., 2021).

Hemicellulose is composed of polysaccharides with β-1,4-linked backbones of xylose, mannose, and glucose (Malinovsky et al., 2014). Changes in the content and the acetylation of hemicellulose in the cell wall confer plants resistance to phytopathogenic microbes (Bacete et al., 2018). In contrast to wild-type plants, *Arabidopsis det3* and *irx6* mutants with increased levels of xylose confer enhanced resistance to the fungus *Plectosphaerella cucumerina* (Brown et al., 2005; Wan et al., 2021). Sufficient evidence suggests that the degree of xylan acetylation affects the resistance of plants to fungal and bacterial pathogens (Shi et al., 2023). The *Arabidopsis* mutant *rwa2* with decreased levels of xylan acetylation exhibits enhanced tolerance to *B. cinerea* (Manabe et al., 2011). Once pathogens breach the cutin layer in plants, pectin functions as a barrier to impede invasion (Wan et al., 2021). The altered pectin biosynthetic pathway in *Arabidopsis thaliana* results in the susceptibility of plants to *P. syringae* and *B. cinerea* (Zhang et al., 2016). It is suggested that changes in the pectin content or its modification plays a crucial role in plant resistance to pathogenic attacks (Bacete et al., 2018). Pectin methylesterases (PMEs), whose activity is controlled by protein inhibitors (pectin methylesterase inhibitors, PMEIs), regulate the degree of pectin methyl esterification (Lionetti et al., 2017). A highly methylated pectin is associated with strong tolerance to CWDEs (Wan et al., 2021). Immunological staining revealed that the *R. solanacearum*-resistant Hawaii 7996 cultivar exhibits a higher degree of HGA methyl esterification compared with the susceptible cultivar Wva700 (Wydra and Beri, 2006). The overexpression of PMEIs confers plants enhanced resistance to pathogens (Shi et al., 2023). The silencing of CaPMEI1, which encodes a PMEI protein, increases the susceptibility of pepper to *X. campestris* pv. *vesicatoria* (An et al., 2008). Polygalacturonases (PGs) depolymerize the HGA, thereby compromising the CWI. OGAs are released from the HGA backbone and function as elicitors to trigger the plant defense responses (Malinovsky et al., 2014). The wall-associated kinase 1 (WAK1) in *A. thaliana* has been identified as the OGA receptor, suggesting an OGA-induced immunity (Brutus et al., 2010). There are a few demonstrations of the involvement of pectin acetylation in plant biotic stresses. When pectin acetylesterases (CsPAEs) are silenced, transgenic citrus plants show increased resistance to bacterial canker disease (Li et al., 2020).

Lignification is capable of increasing the mechanical strength of the plant cell wall and improving the resistance of plants to CWDE-secreting pathogens (Kashyap et al., 2022). The overexpression of genes involved in lignin biosynthesis confers tomato resistance to bacterial wilt disease (Kashyap et al., 2022). Transcriptomic analysis indicated that the lignin biosynthesis genes of tomato are upregulated upon infection with *R. solanacearum*, suggesting a relationship between *R. solanacearum* resistance and lignin biosynthesis (Ishihara et al., 2012). When the phenylalanine ammonia lyase gene (*PAL1*) is knocked out, the mutant exhibits a significant reduction in lignin accumulation and a weakened resistance to *P. syringae* (Rohde et al., 2004; Huang et al., 2010). The knockout of the transcription factor *MYB15* results in decreased levels of lignin, thereby enhancing the susceptibility of *Arabidopsis* to *P. syringae* (Chezem et al., 2017). There are a few examples showing that an increased lignin content in the cell wall renders plants susceptible to pathogens. The NAC transcription factor RD26 positively regulates the resistance of *Arabidopsis* against *R. solanacearum* by inhibiting lignin biosynthesis (Wang et al., 2025). A reduced lignin content is found in GhMYB4-overexpressing cotton, enhancing the plant resistance to *V. dahliae* (Xiao et al., 2021). This is explained by the decreased lignification changing the CWI and amplifying the release of OGAs, thereby strengthening plant immunity. Suberin is a lipid–phenolic heteropolyester that is deposited between the plasma membrane and the cell wall (Shi et al., 2023). In addition to mitigating water loss, suberin acts as a barrier restricting the horizontal colonization of pathogens (Kashyap et al., 2022). The involvement of abscisic acid (ABA) and ET in suberin formation highlights a significant correlation between plant phytohormones and immune responses (Cottle and Kolattukudy, 1982; Leśniewska et al., 2017). Suberin, as a vascular coating, is induced upon infection with *R. solanacearum*, thereby impeding the spread of pathogens in tomato plants (Shi et al., 2023). Suberin deposition in the xylem vessels functions as a barrier that contributes to pathogenic resistance (Kashyap et al., 2022). For example, suberin reinforcement in paravascular parenchyma cells prevents the movement of the fungus *P. chlamydospora* from one vessel to the adjacent vessel (Pouzoulet et al., 2013, 2017).

## 5 Plant metabolites involved in the resistance against *R. solanacearum*

Plants produce a variety of secondary metabolites that act as protectors inhibiting pathogenic growth and reproduction (Yang et al., 2021b). The metabolites known as phytoalexins exhibit diverse structures and antimicrobial activities (Kumar et al., 2023). The phytoalexins include alkaloids, isoprenoids, and phenylpropanoids (Kumar et al., 2023). In tobacco, coumarin diminishes the activity of acyl homoserine lactone, antagonizes the regulatory proteins of quorum sensing (QS), and eventually restricts the adhesion and colonization of *R. solanacearum* (Qais et al., 2021). The plant secondary metabolite daphnetin weakens the virulence of *R. solanacearum* in tobacco through inhibiting the EPS production and biofilm formation (Yang et al., 2021b). 6-Methylcoumarin functions as an antibacterial metabolite by disrupting the cell division of *R. solanacearum* (Yang et al., 2021a). Three root exudates from mulberry plants, including erucamide, oleamide, and camphor bromide, inhibit the growth of *R. Solanacearum* by inducing oxidative stress (Li et al., 2024). Plant-derived hydroxycoumarins are recognized as phytoalexins that defend against attacks from *R. solanacearum* (Yang et al., 2016). Caffeic acid derived from root exudates inhibits the biofilm formation of *R. solanacearum* through repressing the expression of the *lecM* and *epsE* genes (Li et al., 2021). In addition to its antimicrobial activity, caffeic acid effectively activates phenylalanine ammonia lyase (PAL) and peroxidase (POD), which subsequently regulate the accumulation of lignin and hydroxyproline (Li et al., 2021). Caffeic acid is therefore engineered as a potential and effective antibacterial agent for the control of bacterial wilt disease. Biochemical analysis showed that phytoalexins exhibit various antibacterial activities such as inhibition of biofilm and formation of EPS, damage to the cell wall, and disruption of bacterial cell division.

Aside from phytoalexins, plants combat *R. solanacearum* by reducing the production of metabolites used for pathogen virulence (Shen et al., 2020). *R. solanacearum* utilizes plant-derived metabolites to promote the production of virulent factors (Shen et al., 2020). *R. solanacearum*-resistant tomato varieties impede bacterial reproduction by diminishing the production of l-glutamic acid (Shen et al., 2020). Metabolites in the xylem sap that are required as carbon or nitrogen sources for *R. solanacearum* growth, such as putrescine, alpha-d-glucopyranoside, and arabinitol, are significantly decreased in the resistant cultivar K326, suggesting a defense strategy of the plant against *R. solanacearum* (Yang et al., 2022).

## 6 Discussion

*R. solanacearum*-resistant plants predominantly depend on inducible structural barriers. These barriers, which are activated in response to *R. solanacearum* infection, include the formation of tyloses, gels, and callose and the reinforcement of the cell wall. These defense responses in *R. solanacearum*-resistant plants are primarily regulated by phytohormones, particularly SA, JA, and ET. ET acts synergistically with JA to promote the formation of tyloses in xylem vessels, whereas SA abolishes the JA-induced formation of tyloses. Furthermore, ET and JA play a significant role in reinforcing the cell walls deposited with lignin and suberin (Kashyap et al., 2021; Wan et al., 2021).

In contrast to that in resistant plants, the bacteria proliferate more rapidly within the xylem vessels of susceptible plants. The timely establishment of these barriers is critically important for confining the bacteria at the infected sites. In addition, plants use secondary metabolites as part of their resistance strategy against *R. solanacearum*. Physicochemical defense responses, both vertical and horizontal, are key strategies employed by plants to prevent bacterial wilt disease (**Figure 2**). Horizontal reinforcement of the cell wall and vascular coating with lignin and suberin restrict the bacterial movement between xylem vessels, thereby preventing plant wilting. Furthermore, plants resistant to *R. solanacearum* exhibit morphological changes, suggesting that constitutive barriers contribute to resistance to the pathogen. As crop yield often decreases in highly resistant cultivars due to resource allocation trade-offs, plant molecular biologists frequently release resistant germplasms. The efficient utilization of resistant traits is crucial for the development of *R. solanacearum*-resistant cultivars. The CRISPR-Cas9 technology has been efficiently employed to produce transgenic crops resistant to *R. solanacearum*, including tomato, peanut, and potato (Kashyap et al., 2022). The cultivation of resistant cultivars is an economical and effective strategy to mitigate bacterial wilt disease. For instance, tomato plants expressing the *NPR1* gene from *A. thaliana* exhibit enhanced resistance to bacterial wilt disease (Lin et al., 2004). Moreover, grafting the scion of crop onto an *R. solanacearum*-resistant rootstock represents an effective management strategy for the control of bacterial wilt disease. Understanding the resistance mechanisms employed by plants against *R. solanacearum* will not only provide valuable insights for related research but also facilitate the breeding of *R. solanacearum*-resistant cultivars.
